# Funding the Training of Future Health Services Researchers

**DOI:** 10.1111/1475-6773.12844

**Published:** 2018-03-06

**Authors:** Vincent Mor, Paul Wallace

**Affiliations:** ^1^ Department of Health Services for Policy and Practice School of Public Health Brown University Providence RI; ^2^ AcademyHealth Washington DC

## Perspective and Purpose

*Health Services Research (HSR)* per AcademyHealth is the *“multidisciplinary field of scientific investigation that studies how social factors, financing systems, organizational structures and processes, health technologies, and personal behaviors affect access to health care, the quality and cost of health care, and ultimately our health and well‐being. Its research domains are individuals, families, organizations, institutions, communities, and populations.”* Importantly, the professional role and training of who does *HSR*, the setting and financing for *HSR* training and work, and how results are communicated are unstated. Consequently, the field can include various providers and users of *HSR*, as reflected by other papers in this series.

Rich and Collins (current issue) note a growing range of organizations employing health service researchers (HSRers) and recruiting more. This demand includes academia, care delivery organizations, nonpartisan policy research organizations, and a range of nonprofit and for profit commercial entities providing tools and analysis. These employers reflect varied business models, expectations of HSRers, and applications of *HSR* in meeting business goals. Furthermore, *HSR* outputs may be published in the peer‐reviewed or the grey literature, and/or used for internal purposes.

In examining the current supply of HSRers, Frogner (current issue) analyzed a narrower slice of the field, identifying HSRers participating in traditionally academic outlets such as authorship of peer‐reviewed publication and presentations at national research meetings. While the analysis found a modest increase in supply, it was not designed to look across requirements for the more broadly described field per Rich and Collins (current issue).

We have experienced a range of *HSR* roles and responsibilities. One of us (VM) is a PhD with teaching, mentoring, and professional collaborations across *HSR*, long‐standing funding as an investigator and HSR training program director, and consulting relationships with government, not‐for‐profits and commercial entities. The other (PW) is an MD and *HSR* user in roles with integrated care delivery, policy development, consulting, and design efforts for big data and the learning health system. We both have been active members and board directors of AcademyHealth and related HSR groups.

The purpose of this commentary is to describe the history, current status, and opportunities for funding of *HSR* training. It is intended as a foundation and stimulus for further thinking and innovation among policy makers, program leaders, and those on the ground who learn and teach *HSR*. It is a cross‐walk of limited empiric data with our personal perspectives within the field. We summarize past and available funding, largely targeted for those on academic trajectories. We also address emerging resources that can complement and extend traditional training and support for HSRer professional development.

## Traditional Training of *HSR* Scientists

Training for most aspiring HSRers is initially centered on degree‐oriented programs funded through a combination of federal support, private grants, and personal tuition. For nonclinicians, predoctoral degree training at the masters and doctoral levels is often funded by government fellowships, publicly and privately funded research assistantships, and University‐sponsored teaching assistantships. Postdoctoral training, especially but not exclusively for scientists headed for academic roles, is similarly funded from government, private, and internal University funds.

Clinicians, after attaining a degree such as an MD or BSN/MSN, may receive formal research training during salaried postdoctoral fellowships in a clinical discipline or in an additional degree program. Both clinicians and nonclinicians also may develop formally mentored relationships with other investigators. Finally, early‐career awards for clinicians and nonclinicians who are clearly committed to academic research careers are almost exclusively supported by government.

## Federal Funding for *HSR* Training

The National Institutes of Health (NIH) has been funding institutional and individual graduate and postgraduate *HSR* training programs for decades. Since 2002, all these programs have been renamed for Ruth L. Kirschstein. In addition, the original National Center for HSR (NCHSR) initiated funding for pre‐ and postdoctoral training in *HSR*, which continues through its successor, the Agency for Health Research and Quality (AHRQ). NIH also broadly funds health scientists’ predoctoral and postdoctoral training through the National Research Service Award (NRSA) mechanism. However, for this analysis, as it is not possible to differentiate *HSR* from other disciplines among NIH‐supported trainees, only NRSA funds allocated to the AHRQ are considered devoted to training health scientists in *HSR*.

Table 1 presents funding levels for AHRQ‐funded, early‐year *HSR* training between 2010 and 2015 exclusive of ARRA funding. Funding for individual, predoctoral, and postdoctoral institutional programs has been steadily declining over the period from over $10 million to less than $7.5 million. Similarly, the “K” awards for junior faculty career development have declined by more than half from nearly $10 million to less than $5 million. Only dissertation grants have increased by around 50 percent from $.5 million to around $.75 million. We do not have data on the actual number of trainees supported by this investment—a survey of all individuals in *HSR*‐related programs to determine the proportion receiving federal and other support was beyond the scope of this project.

**Table 1:** Funding Levels for AHRQ Programs, 2010–2015^a^

Program	FY10	FY11	FY12	FY13	FY14	FY15
NRSA (F31, F32, T32)	10,236,025	9,036,378	8,746,299	7,362,000	7,492,907	7,438,859
AHRQ K (K01, K02, K08)	9,802,895	8,260,540	8,561,891	7,773,141	6,599,625	4,934,249
AHRQ R36	532,966	924,956	550,290	849,773	751,992	786,968

a Approximate total funding amounts from FY10‐FY15 for both new and continuing training grants per Harry T. Kwon, Director, Division of Research Education, Office of Extramural Research, Education, and Priority Populations; Agency for Health care Research and Quality [accessed September 15, 2016].

Many graduate *HSR* students are also supported by faculty research grants during their academic training and careers. NIH grants have provisions for paying university tuition up to the same level as supported under a NRSA fellowship; non‐NIH grants and contracts vary with respect to the extent to which tuition is an allowable expense. There are also provisions for supporting postdoctoral research scientists outside the context of institutional postdoctoral training grants and contracts, but there is no current systematic way of determining how many such individuals there are in University and Academic Medical Centers engaged in *HSR*.

Dissertation grants (R36), available from AHRQ and NIH, offer support to graduate students in completing their dissertations. While AHRQ funds between 10 and 15 dissertation grants per cycle, it is unknown how many *HSR*‐related dissertation grants are funded by NIH.

Support for training in patient‐centered outcomes research (PCOR) includes Institutional K‐12 programs funded by AHRQ as well as comparable programs funded by NCATS through the Center for Advancing Translational Sciences (CTSAs). These awards focus on interdisciplinary, early‐career investigators acquiring additional skills to become independently funded scientists. The National Library of Medicine's *hsrinfo* service and associated website has identified several other government‐funded fellowships designed for a diverse set of junior *HSR* investigators (U.S. Library of Medicine 2017).

## Postdegree Support for *HSR* Training

Training of clinicians as *HSR* investigators often utilizes complementary organizational and public funding. The integration of research and clinical training may begin at the predoctoral level, but typically an additional investment of time and dollars will be required in the postdoctoral and early‐career development phases for clinician researchers. Possible paths include organization‐sponsored combined degree programs, individual pursuit of an additional research‐oriented masters or doctoral degree, and participation in a clinically oriented postresidency fellowship program such as in general internal medicine that integrates formal research training up to and including achievement of an additional degree. Public funding of primary care research training is among the priorities of the Health Resources and Services Administration (HRSA 2016). Finally, a postentry‐level clinician may participate in career development programs, such as the Veterans Administration Career Development Program focused on early‐career refinement of research capabilities. The Veterans Administration also supports interdisciplinary postdoctoral trainees in *HSR* and Medical Informatics (U.S. Department of Veterans Affairs 2017).

The privately funded Robert Wood Johnson Foundation (RWJF) Clinical Scholars program provided postdoctoral and early‐career training for about 1,000 physicians from multiple specialties over more than 40 years through 2015. The program provided training in quantitative and qualitative *HSR* methods (RWJF Clinical Scholars 2014). Beginning in 2016, a successor program, the National Clinician Scholars Program, involving the prior academic sites and with new financial support including the Veterans Administration has been created, with 28 physician and nurse scholars in the 2016 cohort (National Clinician Scholars Program 2017). In 2016, the RWJF redesigned and refocused its investment in leadership, with three programs emphasizing collaborative team approaches to complex and persistent challenges to advancing a Culture of Health, including a redesigned Clinical Scholars program, the Interdisciplinary Research Leadership program, and the Culture of Health Leaders program (Robert Wood Johnson Foundation 2017). In addition, they launched the Health Policy Research Scholars program, a new program for the leadership development for first‐ and second‐year full‐time doctoral students from underrepresented populations and/or disadvantaged backgrounds (RWJF Health Policy Research Scholars 2017).

## Supply‐side Adaptation to the Funding Environment

Less than half of the 44 predoctoral and 22 postdoctoral *HSR* graduate training programs listed on the AcademyHealth Training Directory have AHRQ institutional training grants. It follows that many trainees are being supported by a variety of different approaches, such as inclusion of trainees on Faculty grants as noted previously. A systematic survey of these programs was beyond the scope of this project, but the authors are aware of additional innovations, including:

- Placement of predoctoral students into health systems for support as Research Assistants
- Placement of trainees in health care systems with joint supervision by faculty mentors alongside clinicians and quality managers. However, many university graduate programs require tuition even for students who have completed their mandatory residence and their course work—health systems hesitate to pay both the stipend and tuition rather than contracting for a postdoctoral fellow for the same funding.
- Internships in state government agencies such as Departments of Public Health, the Medicaid Agency, or agencies managing states’ health insurance exchanges
- Collaborations with the outcomes divisions of pharmaceutical companies

## Delivery System *HSR* Training opportunities

A hallmark of the prevailing academic model of *HSR* training is the production of independent scientists who compete for investigator‐initiated grants reflecting their interests, with analyses designed to yield definitive causal relationships. As noted by Rich and Collins, the questions health system leadership need to be answered are often more pragmatic and demand more rapid response times than conventional *HSR* would allow. Similarly, a recent PCORI/NAM survey of senior executives from health systems found the speed and focus of current research “out of synch” with fast‐paced changes in the delivery system (Johnson et al. 2014).

We see an opportunity to bridge this gap by offering more firmly embedded *HSR* training including collaborative funding in the applied health care system setting. The following features would position trainees with a greater appreciation of the problem‐solving skills that are critical for a health care system:

- *Predoctoral* trainees after completing their methods and basic course work could be placed as research assistants in a health care system for one to two years with joint supervision by graduate program faculty and health system research or quality improvement senior staff.
- *Postdoctoral* trainees could be paid investigators with negotiated joint supervision by the academic program and the appropriate research or quality improvement group in the health system.
- *Career development training* can be facilitated in the context of a health system that has an established relationship with academic departments that can legitimize the health system research in the eyes of research proposal reviewers.

As a real‐time example of this approach, the AcademyHealth‐sponsored Delivery System Science Fellowship (DSSF) program represents a partnership among a dozen delivery systems. The DSSF provides a paid postdoctoral learning experience for early‐career researchers. Host sites and AcademyHealth collaborate to identify and evaluate candidates. The delivery organizations then recruit individual fellows based on their personal preferences and recommendations from the review committee. Sponsoring sites provide the fellows’ full‐time salary and benefits for a minimum of one year as well as travel and registration support to attend AcademyHealth's Annual Research Meeting. Projects are dictated by the needs of the host site and are largely intended for public dissemination or peer review. To date, 118 individuals have applied and 25 fellows, six trained as physicians, have been accepted. Twenty‐one have completed the fellowship and four are current fellows. Fellows have a wide range of backgrounds, including epidemiology, exercise physiology, health psychology, anthropology, clinical medicine, qualitative methods, organizational behavior, and systems engineering (Kanani et al. 2017).

Analogous postdegree training, as noted by Rich and Collins and consistent with our experience, also occurs on other HSRer career paths, emphasizing the skills needed to adapt primary *HSR* capabilities to other business environments. For example, an individual entering a nonpartisan policy research organization will need robust skills for proposal development, project management, and team oversight that may not be experienced in a traditional academic track. Similarly, a scientist new to the R&D environment of a Life Sciences company will need skills around placing *HSR* in the context of a business plan and competing for internal product‐focused research budgets.

## Summary

Direct government support for *HSR* training has plateaued (or declined) while the number and variety of organizations seeking HSRers as problem solvers are expanding. The other papers in this series illustrate a consequent tension between a relatively stable stock of HSRers in academic roles juxtaposed with expanding demand and changing expectations from other settings. We are cautiously optimistic that the growing engagement of demand‐side sectors will balance if not expand the overall funding of opportunities for HSRers.

With the demand for *HSR* expanding across this more varied set of users, we would also project that challenges for the field and its professional development will include the following: (1) how topics for investigation are formulated and prioritized; (2) whether problems will be addressed by solitary investigators or more broadly defined teams; (3) how new data sources, analytic methods, and the need for more timely results will affect the supply and demand for *HSR* work; (4) how shifts in support from largely publicly funded to institutionally supported research may influence the generalizability of discoveries; and (5) how proprietary concerns regarding data, predictive models, and study results can be balanced against the public good of broad dissemination.

Finally, we believe that the key change needed to productively address the above challenges will be a closer collaboration between *HSR* users, especially health systems, and academic *HSR* training programs to work towards producing timely, internally relevant, and externally generalizable knowledge. A sustainable solution will require active and ongoing dialog between those who produce *HSR* and those who consume and likely will increasingly directly pay for it. In this changing environment lies a major opportunity to systematically address the pragmatic research interests of those served by *HSR* and to support expansion of *HSR* training opportunities.

## Supporting information

Appendix SA1: Author Matrix.
